# Exploring Multiple Strategic Problem Solving Behaviors in Educational Psychology Research by Using Mixture Cognitive Diagnosis Model

**DOI:** 10.3389/fpsyg.2021.568348

**Published:** 2021-06-03

**Authors:** Jiwei Zhang, Jing Lu, Jing Yang, Zhaoyuan Zhang, Shanshan Sun

**Affiliations:** ^1^Key Lab of Statistical Modeling and Data Analysis of Yunnan Province, School of Mathematics and Statistics, Yunnan University, Kunming, China; ^2^Key Laboratory of Applied Statistics of MOE, School of Mathematics and Statistics, Northeast Normal University, Changchun, China; ^3^College of Mathematics, Taiyuan University of Technology, Jinzhong, China; ^4^School of Mathematics and Statistics, Yili Normal University, Yili, China; ^5^Government of Jilin Province, Changchun, China

**Keywords:** Bayesian inference, cognitive diagnosis, classification, Markov chain Monte Carlo, multiple-strategy models

## Abstract

A mixture cognitive diagnosis model (CDM), which is called mixture multiple strategy-Deterministic, Inputs, Noisy “and” Gate (MMS-DINA) model, is proposed to investigate individual differences in the selection of response categories in multiple-strategy items. The MMS-DINA model system is an effective psychometric and statistical approach consisting of multiple strategies for practical skills diagnostic testing, which not only allows for multiple strategies of problem solving, but also allows for different strategies to be associated with different levels of difficulty. A Markov chain Monte Carlo (MCMC) algorithm for parameter estimation is given to estimate model, and four simulation studies are presented to evaluate the performance of the MCMC algorithm. Based on the available MCMC outputs, two Bayesian model selection criteria are computed for guiding the choice of the single strategy DINA model and multiple strategy DINA models. An analysis of fraction subtraction data is provided as an illustration example.

## 1. Introduction

Multiple classification latent class models, namely cognitive diagnosis models (CDMs), have been developed specifically to diagnose the presence or absence of multiple fine-grained skills required for solving problems in an examination (Doignon and Falmagne, 1999; Junker and Sijtsma, 2001; Tatsuoka, 2002; de la Torre and Douglas, 2004; Templin and Henson, 2006; DiBello et al., 2007; Haberman and von Davier, 2007; de la Torre, 2009, 2011; Henson et al., 2009; von Davier, 2014; Chen et al., 2015). Compared with the traditional item response theory models, one of the advantages of multiple classification latent class models is that they can provide effective measurement of student learning and progression, design better teaching instruction, and conduct possibly intervention guidance for different individual and group needs.

However, most CDMs only consider the probability that examinees solve a problem in one way. In fact, examinees may solve a problem in different ways. Fuson et al. (1997) found that the children at elementary schools used more than one strategy to solve the problem of multi-digit addition and subtraction. Moreover, in eye-movement studies, Gorin (2007) expounded that the subjects often used very different cognitive strategies when solving similar reading tasks. More specifically, an example of a multiple-strategy used by de la Torre and Douglas (2008) in educational research is on the analysis of fraction subtraction data including responses of 2,144 examinees to 15 fraction subtraction items. The attributes required for the fraction subtraction are as follows: (a) performing basic fraction subtraction operation; (b) simplifying/reducing; (c) separating whole number from fraction; (d) borrowing one from whole number to fraction; (e) converting whole number to fraction; (f) converting mixed number to fraction; (g) column borrowing in subtraction (de la Torre and Douglas, 2008). As an illustration, they use two strategies to solve $4\frac{4}{12}-2\frac{7}{12}$. Strategy 1 requires attributes a, b, c, and d. Strategy 2 requires attributes a, b, and f. The detailed calculation processes were shown in de la Torre and Douglas (2008).

de la Torre and Douglas (2008) proposed a multiple strategy-Deterministic, Inputs, Noisy “and” Gate (MS-DINA) model to address the problem of fraction subtraction, where the DINA model (Haertel, 1989; Doignon and Falmagne, 1999; Junker and Sijtsma, 2001; de la Torre and Douglas, 2004; de la Torre, 2009) was the most popular and widely used model among various CDMs which assumed that examinees were expected to answer an item correctly only when they possessed all the required attributes. The MS-DINA model is a straightforward extension of the DINA model that allows incorporating multiple strategies for cognitive diagnosis based on competing assumptions. However, as de la Torre and Douglas (2008) indicated, although the simplicity of the MS-DINA model was appealing, it made a restrictive assumption that the item parameters were same for different strategies, which implied that the application of each strategy was equally difficulty. Another limitation of MS-DINA model is that the joint distribution attributes is expressed as a function of a higher-order continuous ability. The joint distribution of the attributes as the most special form of the saturated model may not be applied to all cases (Huo and de la Torre, 2014). Moreover, the MS-DINA model cannot provide the information of the strategies selected by the examinees, that is, in the case that multiple strategies are available, the probability of each strategy being used cannot be obtained, and the strategy diagnosis for examinees is an important part in the multiple strategies cognitive diagnosis.

To maximize the diagnostic results of multiple-strategy (MS) assessment and overcome the limitation that assumes identical item parameters across strategies, in this paper, we propose a cognitive diagnosis framework for analyzing the MS data. Specifically, the framework describes a psychometric model that can exploit multiple-strategy information. The psychometric model is a multiple-strategy model called the mixture multiple-strategy DINA (MMS-DINA) model. The details of the framework are laid out in section 2. In section 3, MCMC algorithm is employed to estimate model parameters. In section 4, four simulation studies are used to evaluate the viability of the proposed framework and to simulate true testing conditions to evaluate the performance of the MCMC algorithm based on several different criteria. According to the available MCMC outputs, two Bayesian model selection criteria are computed to guide the choice of the single strategy DINA model and multiple strategy DINA models. An empirical example of fraction subtraction is used to illustrate the application of the proposed MMS-DINA model in section 5. The final section concludes the article with discussion and some directions for further research.

## 2. Models

### 2.1. Multiple-Strategy DINA Model

The MS-DINA model (de la Torre and Douglas, 2008; Huo and de la Torre, 2014) is a straightforward extension of the DINA model, which allows several different strategies of solution for each item. Let *u*_*ij*_ denote the observed item response for the *i*th examinee to response *j*th item, where *i* = 1, 2, …, *N*, and *j* = 1, 2, …, *J*, *u*_*ij*_ = 1, if the *i*th examinee correct answer the *j*th item, 0 otherwise. The *i*th examinee mastery attribute profile, ***α***_*i*_, can be represented by a vector of length *K*, that is, $\alpha_{i}={\left(\alpha_{i1},\alpha_{i2},\ldots ,\alpha_{ik},\ldots ,\alpha_{iK}\right)}^{{}^{\prime }}$, where

$$\alpha_{ik}=\left\{ \begin{array}{l} 1,\text{ the }i\text{th examinee masters the }k\text{th attribute;}\\ \text{0, otherwise.} \end{array}\right.$$

Suppose each item has as many as *M* distinct strategies that would suffice to solve it. A strategy is defined as a subset of the *K* attributes which could be used together to solve the item. This may be coded by constructing *M* different matrices, *Q*_1_, …, *Q*_*M*_, and the element in the *j*th row and *k*th column of *Q*_*m*_(*m* = 1, 2, …, *M*) is denoted as

$$q_{jkm}=\left\{ \begin{array}{l} 1,\text{ if item }j\text{ requires skill }k\text{ of }m\text{th strategy}\\ \text{0, otherwise} \end{array}\right.$$

Let

$$\eta_{ijm}=\overset{K}{\underset{k=1}{\prod }}\alpha_{ik}^{q_{jkm}},m=1,2,\ldots ,M.$$

The latent variable η_*ijm*_ denotes whether the examinee *i* has the all the required attributes to apply the *m*th strategy to the *j*th item. Let

$$\eta_{ij}=\max\left\{\eta_{ij1},\eta_{ij2},\ldots ,\eta_{ijm},\ldots ,\eta_{ijM}\right\}.$$

The variable η_*ij*_ is 1 if examinee *i* satisfies the attribute requirements of at least one of the *M* strategies. Therefore, the item response function of the MS-DINA model is given as

(1) $$\begin{array}{l} p\left(u_{ij}=1|\alpha_{i}\right)={\left(1-s_{j}\right)}^{\eta_{ij}}g_{j}^{1-\eta_{ij}}, \end{array}$$

where the parameter *s*_*j*_ is the slipping parameter, which indicates the probability of slipping on the *j*th item when an examinee has mastered all the required attributes for at least one of the strategies. The parameter *g*_*j*_ is the guessing parameter, which denotes the probability of correctly answering the *j*th item when an examinee does not master all the required attributes for at least one of the strategies.

### 2.2. Mixture Multiple-Strategy DINA Model

We can see that the MS-DINA model assumes that the slipping and guessing parameters are the same for different strategies. The assumption that the application of each strategy has equally difficulty is too restrictive, as indicated by de la Torre and Douglas (2004). Then, de la Torre and Douglas (2008) tried and suggested a variant of the multiple-strategy model in order to break the limitation mentioned above. However, one of the issues they discussed is a feasible approach for estimating the parameters in their model can not be provided due to the necessary identifiability issues. Inspired by their thoughts, we propose a multiple-strategy model to overcome the limitation that assumes identical item parameters across strategies. One way to solve the problem is to use a discrete mixture model. Discrete mixture models assume that a data set is composed of distinct subpopulations of observations that are described by different parametric distributions (Titterington et al., 1985). Thus, a mixture multiple-strategy-DINA (MMS-DINA) model is proposed to allow for different strategies to be associated with different levels of difficulty. The item response function of the MMS-DINA model is given by

(2) $$\begin{array}{l} p\left(u_{ij}=1|\alpha_{i}\right)=\overset{M}{\underset{m=1}{\sum }}\pi_{m}p_{ijm}=\overset{M}{\underset{m=1}{\sum }}\pi_{m}{\left(1-s_{jm}\right)}^{\eta_{ijm}}g_{jm}^{1-\eta_{ijm}}, \end{array}$$

swhere *M* is the number of strategy, *p*_*ijm*_ indicates the correct response probability that the *i*th examinee adopts the *m*th strategy to answer the *j*th item, and π_*m*_(*m* = 1, 2, …, *M*) is a mixing proportion satisfying $\overset{M}{\underset{m=1}{\sum }}\pi_{m}=1.$ In addition to the specific strategy, mixing proportion parameters are related to the distribution of ***α***. The average value of latent attributes for all examinees (***α***) using strategy *m* is μ_*m*_. The parameters *s*_*jm*_ and *g*_*jm*_ denote the slipping and guessing parameters for the *m*th strategy to the *j*th item, respectively. When the number of strategies is one (i.e., *M* = 1), it is apparent that the MMS-DINA model in Equation (2) reduces to the DINA model.

## 3. Bayesian Inferences

### 3.1. Bayesian Estimation

Within a fully Bayesian framework, the Metropolis-Hastings within the Gibbs sampling algorithm (Geman and Geman, 1984; Casella and George, 1992; Chib and Greenberg, 1995; Gilks, 1996; Patz and Junker, 1999a,b) is used to estimate the model parameters. In fact, MCMC methods have been found to be particularly useful in estimating mixture distributions (Diebold and Robert, 1994), including mixtures that involve random effects within classes (Lenk and DeSarbo, 2000). A common MCMC strategy is to sample a class membership parameter for each observation at each stage of the Markov chain (Robert, 1996). For the current model, a strategy membership parameter, *c*_*i*_ = 1, 2, …, *M*, is sampled for each examinee *i* along with a latent attribute parameter ***α***_*i*_. Then, the item response function of the MMS-DINA model in Equation (2) can be expressed as

(3) $$\begin{array}{l} p\left(u_{ij}=1|\alpha_{i},s_{j},g_{j}\right)=\overset{M}{\underset{m=1}{\sum }}p\left(c_{i}=m\right){\left(1-s_{jm}\right)}^{\eta_{ijm}}g_{jm}^{1-\eta_{ijm}}, \end{array}$$

where the latent variable *c*_*i*_ takes a value in the set {1, 2, …, *M*} for the *i*th examinee, indicating which type of strategies the *i*th examinee uses.

The following prior distributions for ***π***, ***c***, ***α***, ***s***, and ***g*** are used in conjunction with the MMS-DINA model, where ***c*** = (*c*_1_, *c*_2_, …, *c*_*N*_), ***s*** = (*s*_1_, *s*_2_, …, *s*_*J*_) and ***g*** = (*g*_1_, *g*_2_, …, *g*_*J*_),

$$\pi =\left(\pi_{1},\pi_{2},\ldots ,\pi_{M}\right)\sim Dirichlet\;\left(\beta_{1},\beta_{2},\ldots ,\beta_{M}\right),$$

$$c_{i}\sim Multinominal\;\left(1|\pi_{1},\pi_{2},\ldots ,\pi_{M}\right),$$

$$\mu_{m}\sim Beta\;\left(\lambda_{1},\lambda_{2}\right),$$

$$\left[\alpha_{ik}|c_{i}=m\right]\sim Bernoulli\;\left(\mu_{m}\right),$$

$$s_{jm}\sim 4\text{-}Beta\;\left(v_{s},t_{s},a_{s},b_{s}\right),$$

$$g_{jm}\sim 4\text{-}Beta\;\left(v_{g},t_{g},a_{g},b_{g}\right).$$

Based on the results of de la Torre and Douglas (2004)'s research, we use the four-parameter *Beta* distribution as the prior distribution of slipping and guessing parameters. The four parameter *Beta* distribution, 4-*Beta* (*v, t, a, b*), is a generalization of the *Beta* (*v, t*) distribution, and it has the interval (*a, b*) rather than (0, 1) as its support set. Then, the joint posterior distribution can be written as

(4) $$\begin{array}{l} p\left(\alpha ,s,g,\pi |u\right)\propto \left[\overset{N}{\underset{i=1}{\prod }}\overset{J}{\underset{j=1}{\prod }}\overset{M}{\underset{m=1}{\sum }}p\left(c_{i}=m\right)f\left(u_{ij}|\alpha_{i},s_{jm},g_{jm}\right)\right]\\ \;\left[\overset{N}{\underset{i=1}{\prod }}p{\left(\alpha_{i}|\mu_{m}\right)}^{\text{I}\left(c_{i}=m\right)}\right]\\ \;\times f_{prior}\left(\mu_{m}\right)\left[\overset{M}{\underset{m=1}{\prod }}\overset{J}{\underset{j=1}{\prod }}f_{prior}\left(s_{jm}\right)f_{prior}\left(g_{jm}\right)\right]\\ \;\overset{M}{\underset{m=1}{\prod }}f_{prior}\left(\pi_{m}\right), \end{array}$$

where $u={\left(u_{1},u_{2},\ldots ,u_{i},\ldots ,u_{N}\right)}^{{}^{\prime }}$ and ***u***_*i*_ = (*u*_*i*1_, *u*_*i*2_, …, *u*_*iJ*_).

The MCMC sampling procedure is composed of the following steps:

**Step 1**: Sample the mixing proportions $\pi ={\left(\pi_{1},s\pi_{2},\ldots ,\pi_{M}\right)}^{{}^{\prime }}.$ Assuming conditional independence between the mixing proportions and all parameters except the strategy memberships of examinees, the mixing proportions have a full condition posterior distribution of the form:

(5) $$\begin{array}{l} p\left(\pi |\text{all other parameters}\right)\propto p\left(c|\pi \right)f_{prior}\left(\pi \right), \end{array}$$

where *n*_*m*_ is the number of examinees using strategy *m*. This full conditional distribution is

$$Dirichlet\left(\beta_{1}+n_{1},\beta_{2}+n_{2},\ldots ,\beta_{M}+n_{M}\right).$$

**Step 2**: Sample a strategy membership *c*_*i*_ for each examinee, where *i* = 1, …, *M*. Assuming independence of examinees, the full condition posterior distribution of *c*_*i*_ can be written as

(6) $$\begin{array}{l} p\left(c_{i}=m|\text{all other parameters}\right)\propto p\left(u_{i}|c_{i}=m,\alpha_{i},s_{m},g_{m}\right)\\ \;p\left(\alpha_{i}|\mu_{m},c_{i}=m\right)\\ \;\propto \left[\overset{J}{\underset{j=1}{\prod }}p_{ijm}^{u_{ij}}{\left(1-p_{ijm}\right)}^{1-u_{ij}}\right]\\ \;\overset{K}{\underset{k=1}{\prod }}Bernoulli\left(\alpha_{ik};\mu_{m}\right)\pi_{m}, \end{array}$$

where $u_{i}={\left(u_{i1},\ldots ,u_{iJ}\right)}^{{}^{\prime }}$ is the item response vector for examinee *i* across items, *J* and *K* are respectively the numbers of item and attribute, and *Bernoulli*(α_*ik*_; μ_*m*_) is the *Bernoulli* density evaluated at α_*ik*_ with parameter μ_*m*_.

**Step 3**: Sample attribute mean μ_*m*_ for each strategy. Assuming the attribute distribution parameters are independent of all parameters expect the attribute vectors for examinees in *m*th strategy, the full conditional distribution of μ_*m*_ can be written as

(7) $$\begin{array}{l} p\left(\mu_{m}|\text{all other parameters}\right)\propto \left[\overset{N}{\underset{i=1}{\prod }}p{\left(\alpha_{i}|\mu_{m}\right)}^{{\text{I}}_{\left(c_{i}=m\right)}}\right]f_{prior}\left(\mu_{m}\right), \end{array}$$

which results in the following full conditional distribution for μ_*m*_:

(8) $$\begin{array}{l} \mu_{m}\sim Beta(\sum_{i=1}^{N}\sum_{k=1}^{K}\alpha_{ik}\text{I}\left(c_{i}=m\right)+\lambda_{1},\left(N\times K\right)\\ \;-\sum_{i=1}^{N}\sum_{k=1}^{K}\alpha_{ik}\text{I}\left(c_{i}=m\right)+\lambda_{2}). \end{array}$$

where I(·) denotes the indicator function. I (*c*_*i*_ = *m*) = 1 if the *i*th examinee choose the *m*th strategy to answer the item, 0 otherwise.

**Step 4**: Sample a latent variable ***α***_*i*_ for each examinee, where *i* = 1, …, *N*. Assuming independence of examinees, the full conditional distribution of ***α***_*i*_ can be written as

(9) $$\begin{array}{l} p\left(\alpha_{i}|\text{all other parameters}\right)\propto p\left(u_{i}|c_{i}=m,\alpha_{i},s_{m},g_{m}\right)\\ \;p\left(\alpha_{i}|\mu_{m},c_{i}=m\right)\\ \;\propto \left[\overset{J}{\underset{j=1}{\prod }}p_{ijm}^{u_{ij}}{\left(1-p_{ijm}\right)}^{1-u_{ij}}\right]\\ \;\overset{K}{\underset{k=1}{\prod }}Bernoulli\left(\alpha_{ik};\mu_{m}\right). \end{array}$$

**Step 5**: Sample item parameters *s*_*jm*_ and *g*_*jm*_ for each strategy and each item. Assuming conditional independence across items, the full conditional distribution of *s*_*jm*_ and *g*_*jm*_ can be written as

(10) $$\begin{array}{l} p\left(s_{jm},g_{jm}|\text{all other parameters}\right)\\ \;\propto \left[\overset{N}{\underset{i=1}{\prod }}p\left(u_{j}|c_{i}=m,\alpha_{i},s_{jm},g_{jm}\right)\right]f_{prior}\left(s_{jm}\right)f_{prior}\left(g_{jm}\right)\\ \;\propto \left\{\overset{N}{\underset{i=1}{\prod }}{\left[p_{ijm}^{u_{ij}}{\left(1-p_{ijm}\right)}^{1-u_{ij}}\right]}^{\text{I}\left(c_{i}=m\right)}\right\}\left[Beta\left(s_{jm};v_{s},t_{s},a_{s},b_{s}\right)\right]\\ \;\times \left[Beta\left(g_{jm};v_{g},t_{g},a_{g},b_{g}\right)\right], \end{array}$$

where $u_{j}={\left(u_{1j},\ldots ,u_{Nj}\right)}^{{}^{\prime }}$ is the item response vector for item *j* across examinees, *N* is the number of examinees.

### 3.2. Bayesian Model Assessment

Within the Bayesian framework, the deviance information criterion (DIC; Spiegelhalter et al., 2002) and the logarithm of the pseudo-marignal likelihood (LPML; Geisser and Eddy, 1979; Ibrahim et al., 2001) are considered to compare three different models (the DINA model, the MS-DINA model, and the MMS-DINA model). As an explanation, we only provide the most complicated calculation process of DIC and LPML in the MMS-DINA model, and the calculation formulas of DIC and LPML for the DINA model and MS-DINA model are similar. These two criteria are based on the log-likelihood functions evaluated at the posterior samples of model parameters. Therefore, the DIC and LPML of the MMS-DINA model can be easily computed. Let **Ω** = (**Ω**_*ij*_, *i* = 1, …, *N, j* = 1, …, *J, m* = 1, …, *M*), where $\Omega_{ijm}={\left(\alpha_{i},\;s_{jm},\;g_{jm},\;\pi_{m}\right)}^{{}^{\prime }}$. Let {**Ω**^(1)^, …, **Ω**^(*R*)^}, where $\Omega^{\left(r\right)}=(\Omega_{ijm}^{\left(r\right)},\;i=1,\ldots ,N,\;j=1,\ldots ,J$,*m* = 1, …, *M*), $\Omega_{ijm}^{\left(r\right)}=\alpha_{i}^{\left(r\right)},s_{jm}^{\left(r\right)},g_{jm}^{\left(r\right)},\pi_{m}^{\left(r\right)}{}^{{}^{\prime }}$ for *i* = 1, …, *N*, *j* = 1, …, *J*, *m* = 1, …, *M* and *r* = 1, …, *R*, which denotes *r*th MCMC sample from the posterior distribution in (4). The joint likelihood function of the responses can be written as

(11) $$\begin{array}{l} L\left(u|\Omega \right)=\overset{N}{\underset{i=1}{\prod }}\overset{J}{\underset{j=1}{\prod }}\overset{M}{\underset{m=1}{\sum }}\pi_{m}p\left(u_{ij}|\alpha_{i},s_{jm},g_{jm}\right), \end{array}$$

where *p* (*u*_*ij*_|***α***_*i*_, *s*_*jm*_, *g*_*jm*_) is the response probability. The logarithm of the joint likelihood function in (11) evaluated at **Ω**^(*r*)^ is given by

(12) $$\begin{array}{l} \log L\left(u|\Omega^{\left(r\right)}\right)=\overset{N}{\underset{i=1}{\sum }}\overset{J}{\underset{j=1}{\sum }}\log\overset{M}{\underset{m=1}{\sum }}\pi_{m}^{\left(r\right)}p\left(u_{ij}|\alpha_{i}^{\left(r\right)},s_{jm}^{\left(r\right)},g_{jm}^{\left(r\right)}\right). \end{array}$$

Since the joint log-likelihoods for the responses, $\log\overset{M}{\underset{m=1}{\sum }}\pi_{m}^{\left(r\right)}p\left(u_{ij}|\alpha_{i}^{\left(r\right)},s_{jm}^{\left(r\right)},g_{jm}^{\left(r\right)}\right)$, *i* = 1, …, *N, j* = 1, …, *J*, and *m* = 1, …, *M* are readily available from MCMC sampling outputs, $\log\overset{M}{\underset{m=1}{\sum }}\pi_{m}^{\left(r\right)}p\left(u_{ij}|\alpha_{i}^{\left(r\right)},s_{jm}^{\left(r\right)},\;g_{jm}^{\left(r\right)}\right)$ in (12) is easy to compute. Now, we calculate DIC as follows

(13) $$\begin{array}{l} \text{DIC}=\hat{\text{Dev}\left(\Omega \right)}+2P_{D}=\hat{\text{Dev}\left(\Omega \right)}+2\left[\bar{\text{Dev}\left(\Omega \right)}-\hat{\text{Dev}\left(\Omega \right)}\right], \end{array}$$

where

$$\begin{array}{l} \bar{\text{Dev}\left(\Omega \right)}=-\frac{2}{R}\overset{R}{\underset{r=1}{\sum }}\log L\left(u|\Omega^{\left(r\right)}\right)\text{ and}\hat{\text{Dev}\left(\Omega \right)}\\ \;=-2\underset{1\leq r\leq R}{\max}\log L\left(u|\Omega^{\left(r\right)}\right). \end{array}$$

In (13), $\bar{\text{Dev}\left(\Omega \right)}$ is a Monte Carlo estimate of the posterior expectation of the deviance function Dev(**Ω**) = −2 log *L*(***u***|**Ω**), $\hat{\text{Dev}\left(\Omega \right)}$ is an approximation of $\text{Dev}\left(\hat{\Omega }\right),$ where $\hat{\Omega }$ is the posterior mode, when the prior is relatively non-informative, and $P_{D}=\bar{\text{Dev}\left(\Omega \right)}-\hat{\text{Dev}\left(\Omega \right)}$ is the effective number of parameters. Based on our construction, both DIC and *P*_*D*_ given in (13) are always non-negative. The model with a smaller DIC value fits the data better.

Letting $G_{ij,\max}=\underset{1\leq r\leq R}{\max}\left[-\log\overset{M}{\underset{m=1}{\sum }}\pi_{m}^{\left(r\right)}p\left(u_{ij}|\alpha_{i}^{\left(r\right)},s_{jm}^{\left(r\right)},g_{jm}^{\left(r\right)}\right)\right]$, a Monte Carlo estimate of the conditional predictive ordinate (CPO; Gelfand et al., 1992; Chen et al., 2000) is given by

(14) $$\begin{array}{l} \log\hat{\left({\text{CPO}}_{ij}\right)}=-G_{ij,\max}\\ \;-\log\;[\frac{1}{R}\sum_{r=1}^{R}\exp\;\{-\log\sum_{m=1}^{M}\pi_{m}^{\left(r\right)}p\left(u_{ij}|\alpha_{i}^{\left(r\right)},s_{jm}^{\left(r\right)},g_{jm}^{\left(r\right)}\right)\\ \;-U_{ij,\max}\}]. \end{array}$$

Note that the maximum value adjustment used in $\log\hat{\left(\text{CP}{\text{O}}_{ij}\right)}$ plays an important role in numerical stabilization in computing $\exp\left\{-\log\overset{M}{\underset{m=1}{\sum }}\pi_{m}^{\left(r\right)}p\left(u_{ij}|\alpha_{i}^{\left(r\right)},s_{jm}^{\left(r\right)},g_{jm}^{\left(r\right)}\right)-G_{ij,\max}\right\}$ in (14). A summary statistic of the $\hat{\text{CP}{\text{O}}_{ij}}$ is the sum of their logarithms, which is called the LPML and given by

(15) $$\begin{array}{l} \text{LPML}=\overset{N}{\underset{i=1}{\sum }}\overset{J}{\underset{j=1}{\sum }}\log\hat{\left(\text{CP}{\text{O}}_{ij}\right)}. \end{array}$$

The model with a larger LPML has a better fit to the data.

### 3.3. The Accuracy Evaluation of Parameter Estimation

To implement the MCMC sampling algorithm, chains of length 10,000 with an initial burn-in period 5,000 are chosen. Fifty replications are used in the following simulation studies. Three indices are used to assess the accuracy of the parameter estimates. Let ϑ be the parameter of interest. Assume that *M* = 50 data sets are generated. Also, let ${\hat{\vartheta }}^{\left(m\right)}$ and SD^(*m*)^(ϑ) denote the posterior mean and the posterior standard deviation of ϑ obtained from the *m*th simulated data set for *m* = 1, …, *M*.

The Bias for parameter ϑ is defined as

(16) $$\begin{array}{l} Bias\left(\vartheta \right)=\frac{1}{M}\overset{M}{\underset{m=1}{\sum }}\left({\hat{\vartheta }}^{\left(m\right)}-\vartheta \right), \end{array}$$

and the mean squared error (MSE) for parameter ϑ is defined as

(17) $$\begin{array}{l} \text{MSE}\left(\vartheta \right)=\frac{1}{M}\overset{M}{\underset{m=1}{\sum }}{\left({\hat{\vartheta }}^{\left(m\right)}-\vartheta \right)}^{2}, \end{array}$$

and the average of posterior standard deviation can be defined as

(18) $$\begin{array}{l} \text{SD}\left(\vartheta \right)=\frac{1}{M}\overset{M}{\underset{m=1}{\sum }}\text{S}{\text{D}}^{\left(m\right)}\left(\vartheta \right). \end{array}$$

In addition, four criteria are used to assess the accuracy of the examinee classification methods. These criteria include the following: (h) the marginal correct classification rate for each attribute; (t) the proportion of examinees classified correctly for all *K* attributes; (v) the proportion of examinees classified correctly for at least *K* − 1 attributes; (z) the proportion of examinees classified incorrectly for *K* − 1 or *K* attributes.

## 4. Simulation

### 4.1. Simulation 1

This simulation study is conducted to evaluate the parameter recoveries of the proposed model using the MCMC algorithm as the number of examinees increases. Here, we fix the test length and the numbers of attributes.

#### 4.1.1. Simulation Designs

The following manipulated conditions are considered. Test length is fixed at 20, and 2 strategies with 5 attributes are used in this simulation. The corresponding *Q* matrix of the 20 items is the same as de la Torre (2008, p. 605); and the number of examinees, *N* = 500, 1, 000, and 2, 000. Fully crossing different levels produce 3 simulation conditions (1 test length × 3 sample sizes). The true values of slipping and guessing parameters are set to be 0.3 and 0.1, respectively. Assuming independence among examinees and independence among attributes, the true value of α_*ik*_ is generated from *Bernoulli*(0.5). We can obtain a *N* × 5 matrix ***α***, where $\alpha ={\left(\alpha_{1},\alpha_{2},\ldots ,\alpha_{i},\ldots ,\alpha_{N}\right)}^{{}^{\prime }},$ and the *i*th row vector ***α***_*i*_ denotes the *i*th examinee's true cognitive state. The hyper-parameters of the prior distributions are fixed as follows: β_1_ = β_2_ = 0.01, and λ_1_ = λ_2_ = 0.5. We assume the priors of the slipping and guessing parameters to follow a 4-*Beta* (1, 2, 0.1, 0.5) based on de la Torre and Douglas (2004)'s paper. Response data are simulated using the MMS-DINA model. About 50 replications are considered to evaluate the parameters recovery in this simulation.

To evaluate the convergence of parameter estimations, we only consider the convergence in the case of minimum sample sizes. That is, the number of examinees is 500. Two methods are used to check the convergence of our algorithm. One is the “eyeball” method to monitor the convergence by visually inspecting the history plots of the generated sequences (Hung and Wang, 2012), and the other method is to use the Gelman-Rubin method (Gelman and Rubin, 1992; Brooks and Gelman, 1998) to check the convergence of the parameters. The convergence of Bayesian algorithm is checked by monitoring the trace plots of the parameters for consecutive sequences of 10,000 iterations. The trace plots show that all parameter estimates converge quickly. We set the first 5,000 iterations as the burn-in period. In addition, the values of the potential scale reduction factor $\hat{R}$ (PSRF; Brooks and Gelman, 1998) are calculated. We find the PSRF (Brooks and Gelman, 1998) values of all parameters are less than 1.2, which ensures that all chains converge as expected.

#### 4.1.2. Recovery Results Based on Minimum Sample Sizes

As an illustration, we only show the Bias, MSE, and SD for all of the slipping and guessing parameters based on 500 examinees. In the case of the strategy 1, the Bias is between 0.083 and 0.110 for the slipping parameters and between 0.053 and 0.096 for the guessing parameters. The MSE is between 0.007 and 0.019 for the slipping parameters and between 0.004 and 0.013 for the guessing parameters. The SD are about 0.057 and 0.020 for the slipping and guessing parameters. In the case of the strategy 2, the Bias is between 0.087 and 0.107 for the slipping parameters and between 0.069 and 0.114 for the guessing parameters. The MSE is between 0.007 and 0.011 for the slipping parameters, between 0.006 and 0.018 for the guessing parameters. The SDs are about 0.057 and 0.022 for the slipping and guessing parameters.

We consider the criteria (h) in this simulation study, and the results show that the marginal correct classification rates are consistently high for the MMS-DINA model. Based on the criteria (t) through (z), we find that the MMS-DINA model consistently classifies examinees correctly high at least *K*−1 attributes and produces few severe misclassifications. Thus, the classification method on the MMS-DINA model is effective.

#### 4.1.3. Item Parameters Recovery Based on Different Sample Sizes

Given the total test length, when the number of individuals increases from 500 to 2,000, the average Bias, MSE, and SD for slipping and guessing parameters decrease. For example, under the first strategy, the average Bias of all slipping parameters decreases from 0.101 to 0.079, the average MSE of all slipping parameters decreases from 0.010 to 0.006, and the average SD of all slipping parameters decreases from 0.057 to 0.044. The average Bias of all guessing parameters decreases from 0.078 to 0.058, the average MSE of all guessing parameters decreases from 0.011 to 0.008, and the average SD of all guessing parameters decreases from 0.021 to 0.016. The evaluation results of the accuracy of item parameter estimation for different numbers of examinees are given in Table 1. We find that as the number of individuals increases, the estimates of item parameters become more accurate. In summary, the estimation of this algorithm is effective and accurate under the condition of simulation study 1.

**Table 1:** Evaluating the accuracy of the item parameters based on different sample sizes in simulation study 1.

	**Strategy 1**							**Strategy 2**					
**Sample**	***s*_**1**_**			***g*_**1**_**				***s*_**2**_**			***g*_**2**_**		
**size**	**ABias**	**AMSE**	**ASD**	**ABias**	**AMSE**	**ASD**		**ABias**	**AMSE**	**ASD**	**ABias**	**AMSE**	**ASD**
500	0.101	0.010	0.057	0.078	0.011	0.021		0.100	0.010	0.057	0.101	0.014	0.022
1,000	0.086	0.007	0.048	0.063	0.009	0.018		0.097	0.009	0.049	0.091	0.010	0.018
2,000	0.079	0.006	0.044	0.058	0.008	0.016		0.089	0.008	0.046	0.083	0.006	0.015

*Note that the ABias, AMSE, and ASD denote the average Bias, average MSE, and average SD for all item parameters*.

### 4.2. Simulation 2

This simulation study is conducted to assess the parameter recoveries of the proposed model using the MCMC algorithm as the number of items increases. Here, we fix the sample size and the numbers of attributes.

#### 4.2.1. Simulation Designs

The following manipulated conditions are considered. The number of examinees is fixed at 1,000, and the number of items, *J* = 20 or 30. Two strategies with five attributes are considered in this simulation. The corresponding *Q* matrix of the 20 items is the same as de la Torre (2008, p. 605), and the *Q* matrix of the 30 items is shown in Table 2. Fully crossing different levels have two conditions (2 test lengths × 1 sample size).

**Table 2:** The *Q* matrix design in simulation 2.

**Item**	**Attribute**									
	**Strategy A**					**Strategy B**				
1	1	1	0	0	0	0	1	0	1	1
2	1	0	1	0	0	0	0	1	1	1
3	1	0	0	1	0	0	1	1	0	1
4	1	0	0	0	1	0	1	1	1	0
5	0	1	1	0	0	1	0	0	1	1
6	0	1	0	1	0	1	1	0	0	1
7	0	1	0	0	1	1	1	0	1	0
8	0	0	1	1	0	1	0	1	0	1
9	0	0	1	0	1	1	0	1	1	0
10	0	0	0	1	1	1	1	1	0	0
11	1	1	1	0	0	0	0	0	1	1
12	1	1	0	1	0	0	1	0	0	1
13	1	1	0	0	1	0	1	0	1	0
14	1	0	1	1	0	0	0	1	0	1
15	1	0	1	0	1	0	0	1	1	0
16	1	0	0	1	1	0	1	1	0	0
17	0	1	1	1	0	1	0	0	0	1
18	0	1	1	0	1	1	0	0	1	0
19	0	1	0	1	1	1	1	0	0	0
20	0	0	1	1	1	1	0	1	0	0
21	1	1	0	0	0	1	0	0	0	0
22	1	0	1	0	0	0	1	0	0	0
23	1	0	0	1	0	0	0	1	0	0
24	1	0	0	0	1	0	0	0	1	0
25	0	1	1	0	0	0	0	0	0	1
26	1	0	0	0	0	0	1	1	0	0
27	0	1	0	0	0	1	0	0	0	1
28	0	0	1	0	0	1	0	0	1	0
29	0	0	0	1	0	1	1	0	0	0
30	0	0	0	0	1	1	0	1	0	0

The true values and prior distributions for the parameters are the same as the simulation 1. To implement the MCMC sampling algorithm, chains of length 10,000 with an initial burn-in period 5,000 are chosen. Fifty replications are considered in this simulation. The following conclusions can be obtained. Given the total number of examinees, when the number of items increases from 20 to 30, the average Bias, MSE, and SD for slipping and guessing parameters increase. For example, for the first strategy, the average Bias of all slipping parameters increases from 0.086 to 0.093, the average MSE of all slipping parameters increases from 0.007 to 0.009, and the average SD of all slipping parameters increases from 0.048 to 0.051. The average Bias of all guessing parameters increases from 0.063 to 0.087, the average MSE of all guessing parameters increases from 0.009 to 0.014, and the average SD of all guessing parameters increases from 0.018 to 0.023. The evaluation results of the accuracy of item parameter estimation for different numbers of items are specified in Table 3.

**Table 3:** Evaluating the accuracy of the item parameters based on different numbers of items in simulation study 2.

	**Strategy 1**					
	***s*_1_**			***g*_1_**		
**Test length**	**ABias**	**AMSE**	**ASD**	**ABias**	**AMSE**	**ASD**
20	0.086	0.007	0.048	0.063	0.009	0.018
30	0.093	0.009	0.051	0.087	0.014	0.023
	**Strategy 2**					
	***s*_2_**			***g*_2_**		
**Test length**	**ABias**	**AMSE**	**ASD**	**ABias**	**AMSE**	**ASD**
20	0.097	0.009	0.049	0.091	0.010	0.018
30	0.106	0.012	0.051	0.103	0.016	0.023

*Note that the ABias, AMSE, and ASD denote the average Bias, average MSE, and average SD for all item parameters*.

### 4.3. Simulation 3

This simulation study is conducted to evaluate the recoveries of the proposed model using the MCMC algorithm as the number of attributes increases. Here, the sample size and the test length are fixed.

#### 4.3.1. Simulation Designs

The following manipulated conditions are considered. The number of examinees is fixed at 1,000, and the number of items is fixed at 40, that is, *J* = 40. Two strategies with seven attributes are considered in this simulation. The corresponding *Q* matrix of the 40 items is shown in Table 4. The true values and prior distributions for the parameters are the same as the simulation 1. To implement the MCMC sampling algorithm, chains of length 10,000 with an initial burn-in period 5,000 are chosen. Fifty replications are considered in this simulation. The recovery results of item parameters are shown in Table 5.

**Table 4:** The Q matrix design in simulation 3.

**Item**	**Attribute**													
	**Strategy A**							**Strategy B**						
1	1	1	1	0	0	0	0	1	1	0	0	0	0	0
2	0	1	0	0	0	0	1	0	1	0	0	0	0	0
3	0	0	1	0	0	0	0	1	0	0	1	0	0	0
4	1	0	0	1	0	0	0	1	0	0	0	1	0	0
5	0	0	1	0	1	0	0	0	0	0	1	0	0	0
6	0	0	0	0	0	1	0	1	0	0	0	0	0	1
7	1	0	0	0	0	0	1	0	0	0	1	1	1	0
8	0	1	0	1	0	0	0	1	0	0	0	0	0	0
9	1	0	1	0	0	0	0	1	0	1	0	0	0	0
10	0	0	0	1	0	0	0	0	0	1	0	0	0	0
11	1	0	0	0	1	0	0	1	0	0	0	0	1	0
12	0	0	1	0	0	1	0	0	0	0	0	1	0	0
13	0	0	0	0	0	0	1	0	0	0	0	0	1	0
14	0	1	1	0	0	0	0	0	0	0	0	0	0	1
15	1	1	0	0	0	0	0	1	1	1	0	0	0	0
16	0	1	0	0	1	0	0	1	1	0	1	0	0	0
17	0	1	0	0	0	0	0	1	1	0	0	1	0	0
18	0	0	1	1	0	0	0	1	1	0	0	0	1	0
19	0	0	0	0	1	0	0	0	0	1	0	0	1	1
20	1	0	0	0	0	1	0	0	1	1	1	0	0	0
21	0	0	1	0	0	0	1	0	0	1	0	1	0	1
22	0	0	0	1	1	0	0	1	1	0	0	0	0	1
23	0	0	0	1	0	1	0	0	1	1	0	0	0	1
24	0	0	0	1	0	1	0	0	0	1	1	1	0	0
25	0	0	0	1	0	0	1	0	0	0	1	0	1	1
26	0	0	0	0	1	1	0	0	1	1	0	0	0	0
27	0	0	0	0	1	0	1	0	0	1	0	0	1	0
28	0	0	0	0	0	1	1	0	1	0	0	1	0	0
29	1	0	0	0	0	0	0	0	1	0	0	0	0	1
30	1	1	0	1	0	0	0	0	0	1	1	0	0	0
31	1	1	0	0	1	0	0	0	0	1	0	1	0	0
32	1	1	0	0	0	1	0	0	1	0	1	0	0	0
33	1	1	0	0	0	0	1	0	0	0	1	0	1	0
34	0	1	1	1	0	0	0	0	0	0	1	1	0	0
35	0	0	1	0	1	0	1	0	0	0	1	0	1	0
36	0	0	1	0	0	1	1	0	0	1	0	0	0	1
37	0	1	1	0	0	0	1	0	0	0	1	0	0	1
38	0	0	1	1	1	0	0	0	0	0	0	1	1	0
39	0	0	0	1	0	1	1	0	0	0	0	1	0	1
40	0	0	0	1	1	1	0	0	0	0	0	0	1	1

**Table 5:** Evaluating the accuracy of the item parameters when the examined attributes increase.

	**Strategy 1**					
	***s*_**1**_**			***g*_**1**_**		
**Test length × Examinee**	**ABias**	**AMSE**	**ASD**	**ABias**	**AMSE**	**ASD**
40 × 1000	0.077	0.008	0.033	0.095	0.015	0.017
	**Strategy 2**					
	***s*_2_**			***g*_2_**		
**Test length × Examinee**	**ABias**	**AMSE**	**ASD**	**ABias**	**AMSE**	**ASD**
40 × 1000	0.085	0.011	0.039	0.097	0.019	0.021

*Note that the ABias, AMSE, and ASD denote the average Bias, average MSE, and average SD for all item parameters*.

We find that when the number of attributes increases, the maximums of the average Bias, MSE, and SD for all of the slipping parameters are 0.085, 0.011, and 0.039, respectively, and the maximums of the average Bias, MSE, and SD for all of the guessing parameters are 0.097, 0.019, and 0.021, respectively. In summary, it is found that the MCMC algorithm can provide accurate parameters and can be used to guide practice through the three different simulation studies.

### 4.4. Simulation 4

In this simulation study, we use the DIC and LPML model assessment criteria to evaluate model fitting.

#### 4.4.1. Simulation Designs

In this simulation, the number of examinees *N* = 1, 000 is considered and the test length is fixed at 20. The *Q* matrix from de la Torre (2008, p. 605)'s paper is used in this simulation study. Three cognitive diagnosis models will be considered. That is, the DINA model, the MS-DINA model, and the MMS-DINA model. Therefore, we evaluate the model fitting in the following three cases.

Case 1: True model: DINA model vs. Fitted model: DINA model, MS-DINA model, and MMS-DINA model;
Case 2: True model: MS-DINA model vs. DINA model, MS-DINA model, and MMS-DINA model;
Case 3: True model: MMS-DINA model vs. Fitted model: DINA model, MS-DINA model, and MMS-DINA model.

The true values and prior distributions for the parameters are the same as the simulation 1. To implement the MCMC sampling algorithm, chains of length 10,000 with an initial burn-in period 5,000 are chosen. The results of the Bayesian model assessment based on the 50 replications are shown in Table 6. Note that the following results of DIC and LPML are based on the average of 50 replications.

**Table 6:** The results of Bayesian model assessment in simulation 4.

	**True Model**		**DINA**	**MS-DINA**	**MMS-DINA**
Fitted	DINA	DIC	**17605.31**	20921.87	20313.99
model		LPML	−**9544.81**	−12047.03	−10336.18
	MS-DINA	DIC	26998.25	**22546.12**	22582.16
		LPML	−13805.85	**−11724.69**	− 13199.05
	MMS-DINA	DIC	21264.30	21023.73	**19944.07**
		LPML	−11851.35	−11393.62	**−10126.66**

*The meaning of the bold values is the model with the best-fitting data among the three candidate models*.

From Table 6, we find that when the DINA model is the true model, the DINA model fits the data best as we expected. The average DIC and LPML for the DINA model are 17605.31 and −9544.81. The second best fitting model is the MMS-DINA model. The differences between DINA model and MMS-DINA model in the average DIC and LPML are −2708.68 and 791.37, respectively. The differences between DINA model and MS-DINA model in the average DIC and LPML are −3316.56 and 2502.22, respectively. This indicates that the MMS-DINA model is more sufficient fitting compared with the MS-DINA model if the data are generated from a simple DINA model. When the MS-DINA model is the true model, the MS-DINA fitting the data generated from the MS-DINA is better than the DINA model and the MMS-DINA model. The DINA model is worst model. The differences between MS-DINA model and MMS-DINA model in the average DIC and LPML are −36.04 and 1474.36, respectively, and the differences between MS-DINA model and DINA model in the average DIC and LPML are −4452.13 and 2081.16, respectively. When the MMS-DINA is the true model, the average DIC difference between MMS-DINA model and MS-DINA (DINA) model is about −1079.66 (−1320.23), and the average LPML difference between MMS-DINA model and MS-DINA (DINA) model is about 1266.96 (1724.69). This shows that when the data come from the mixture multiple strategy model, the DINA model with a single strategy is obviously ineffective in fitting this batch of data. The MS-DINA model has better fitting than the DINA model. No matter which models (DINA and MS-DINA) generate data, the MMS-DINA model is better fitting model than the other not true models. The MMS-DINA model is effective under many conditions of model fitting. In summary, the Bayesian assessment criterion is effective for identifying the true models, and it can be used in the subsequent real data study.

## 5. Empirical Example Analysis

### 5.1. Data

To study the applicability of the mixture multiple-strategy DINA model, we consider a real data including responses by 528 middle school students to answer 15 fraction subtraction items, which is a subset of the data originally used and described by Tatsuoka (2002). The *Q*-matrix design is given in de la Torre and Douglas (2008) research. Two strategies are considered to solve the 15 items, where the attribute definition is the same as in the introduction. The prior distributions described in the simulation section are used for the relevant parameters of the MMS-DINA model. Parameter estimates are based on averaging the estimates from 5 parallel chains with randomly chosen starting values. The standard deviations are obtained by averaging the sample SDs of the parameters from the separate chains. Each of these parallel chains is run for 10,000 iterations with the first 5,000 iterations as burn-in.

### 5.2. Bayesian Model Assessment

Three comparative models, the DINA model, the MS-DINA model, and the MMS-DINA model, are used to fit the fraction subtraction data. The deviance information criterion (DIC; Spiegelhalter et al., 2002) and the logarithm of the pseudo-marignal likelihood (LPML; Geisser and Eddy, 1979; Ibrahim et al., 2001) are computed on the “CODA” R package (Plummer et al., 2006). Based on the comparable values of the DIC, that is, 5941.12 for the DINA model vs. 6652.13 (7306.29) for the MMS-DINA model (MS-DINA model). The LPMLs for the DINA model, MS-DINA model, and the MMS-DINA model are −2970.56, −3653.14, and −3326.06, respectively. The second best fitting model is also the MMS-DINA model. Based on the above model assessment results, we find that the DINA model fits the data most appropriately. The two multiple strategy models may show the over-fitting phenomenon, which results in that the data fitting is not as good as the simple DINA model. In addition, the MMS-DINA model is preferred for this data set because its relatively flexible formulation do not lead to worse fit compared with the MS-DINA model.

### 5.3. Results

The estimated posterior means and the SDs for the MMS-DINA model are shown in Table 7. The estimates of the slipping parameters range from 0.10 to 0.23 and the estimates of the guessing parameters range from 0.10 to 0.25. For the item 2, the students choose two strategies to answer the item, in which the first strategy examines four attributes (attributes 1, 2, 3, and 4), and the second strategy examines two attributes (attributes 1 and 6). We know that the more attributes an item measures, the lower the probability that the specific examinee will answer correctly. This is because the examinee can answer the item correctly if they have mastered all the attributes. If the examinee answers correctly the item with more attributes, the examinee is more likely to guess correctly the item. Therefore, for item 2, the estimate of the guessing parameter under the first strategy is 0.22, which is higher than the estimate of the guessing parameter under the second strategy is 0.18. Similarly, for item 4, the first strategy examines five attributes (attributes 1, 2, 3, 4, and 5) and the second strategy examines three attributes (attributes 1, 5, and 6). The corresponding estimates of guessing parameters are 0.18 and 0.12, respectively. When the number of attributes examine under the two strategies is the same, the estimates of the guessing parameters of the two strategies are basically the same. For example, four attributes are examined under both strategies for item 15. The probability of guessing under both strategies is the same as 0.11. In addition, the three items with the easiest slipping are items 6, 5, and 15 when using the strategy 1, and the corresponding estimates of the slipping parameters are 0.21, 0.20, and 0.17, respectively. When using the strategy 2, the three items with the easiest slipping are items 6, 13, and 2. The corresponding estimates of the slipping parameters are 0.17, 0.15, and 0.14, respectively.

**Table 7:** MMS-DINA model parameter estimates for the fraction subtraction data.

	**Strategy 1**				**Strategy 2**			
	***s***_*****j***1**_		***g***_*****j***1**_		***s***_*****j***2**_		***g***_*****j***2**_	
**Item**	**Estimate**	**SD**	**Estimate**	**SD**	**Estimate**	**SD**	**Estimate**	**SD**
1	0.13	0.02	0.12	0.05	0.13	0.01	0.11	0.01
2	0.12	0.01	0.22	0.03	0.14	0.02	0.18	0.02
3	0.10	0.02	0.20	0.02	0.11	0.02	0.12	0.02
4	0.11	0.03	0.18	0.02	0.13	0.02	0.11	0.02
5	0.20	0.01	0.25	0.03	0.13	0.01	0.23	0.01
6	0.21	0.02	0.10	0.01	0.17	0.02	0.12	0.02
7	0.10	0.01	0.13	0.01	0.13	0.02	0.12	0.02
8	0.10	0.02	0.21	0.03	0.12	0.03	0.14	0.03
9	0.10	0.01	0.19	0.02	0.12	0.01	0.14	0.01
10	0.15	0.03	0.17	0.01	0.12	0.02	0.11	0.02
11	0.15	0.01	0.19	0.02	0.11	0.03	0.12	0.03
12	0.16	0.02	0.12	0.03	0.13	0.01	0.11	0.01
13	0.13	0.03	0.15	0.00	0.15	0.02	0.12	0.02
14	0.15	0.01	0.11	0.01	0.13	0.01	0.12	0.01
15	0.17	0.01	0.11	0.02	0.11	0.02	0.11	0.02

**Figure 1:**
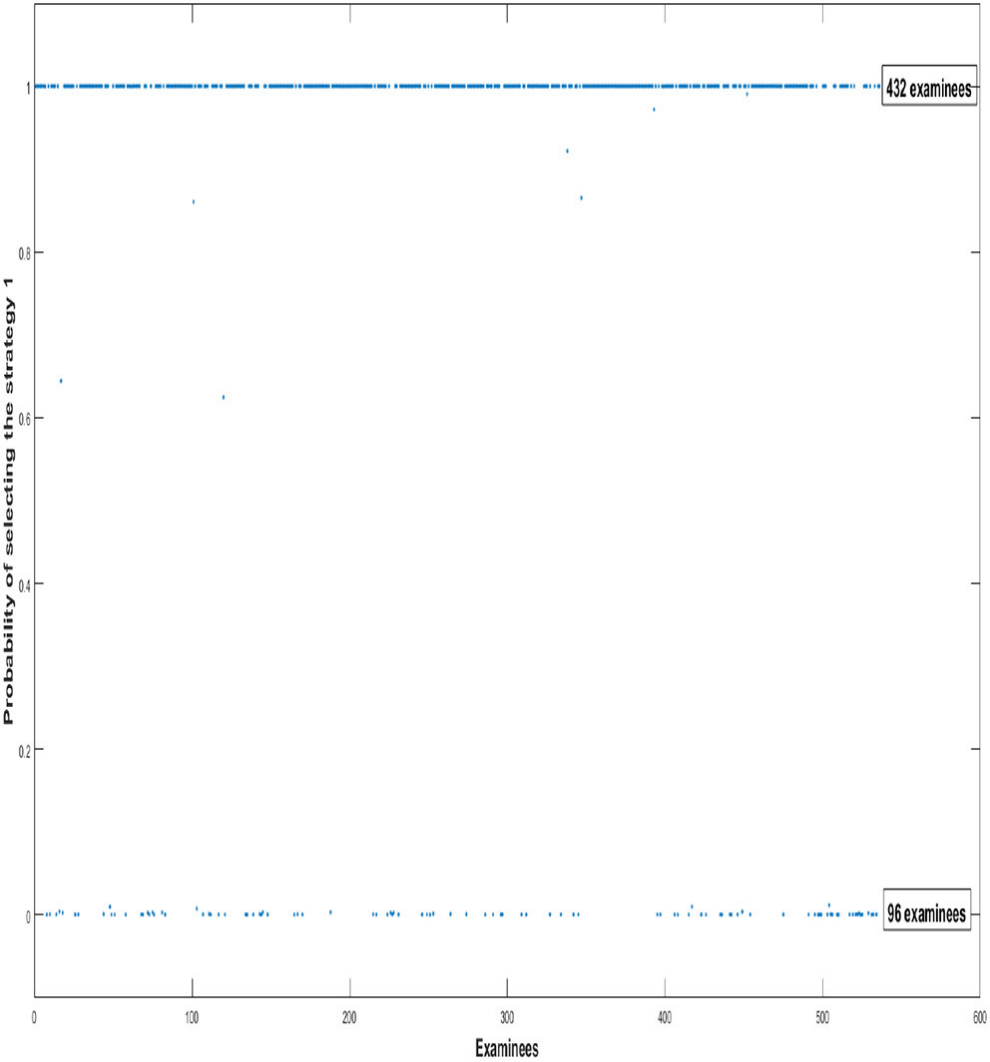
The probabilities of examinees choosing different strategies. The y-axis indicates the probabilities of all examinees using the first strategy to answer items. 0 indicates that examinees use the first strategy to answer item with 0% probability, while 1 indicates that examinees use the first strategy to answer items with 100% probability.

In order to depict individual tendency of which strategy the examinees used, we use the probability plots of examinees choosing different strategies to show the selection tendency of all 528 examinees. In Figure 1, We find that 432 examinees use the first strategy to answer all 15 items. Compared to the first strategy, the number of examinees who adopt the second strategy is relatively small, only 96 examinees.

## 6. Conclusions and Discussion

The goal of this article is to investigate a discrete mixture version of multiple-strategy model for cognitive diagnosis. A unique feature of the mixture model (MMS-DINA model) presented in this article is its capacity to break the limitation that assumes identical item parameters across strategies. The model-based approach presented in this article provides a natural generalization of the DINA model that allows it to account for the strategies to have different item parameters for each item. In the simulation studies, two simulation designs to examine the accuracy of the algorithm estimation from three different perspectives. The simulation results indicate that MCMC algorithm can be used to obtain accurate parameter estimates. Thus, this research provides researchers a tool that allows them to explore the practicability of the MMS-DINA model, which can in turn pave the way for the applications of CDMs in practical education settings to inform instruction and learning. In addition, two Bayesian model assessment criterion are considered to evaluate the model fitting among DINA model, MS-DINA model and MMS-DINA model. We find that when the data are generated from the simple single-strategy DINA model, the MMS-DINA model fits the data better than the MS-DINA model. This may be because each strategy is selected with a certain probability in the MMS-DINA model, unlike the MS-DINA model, which randomly chooses one strategy from multiple strategies. In this way, the *Q* matrix used in the MS-DINA model may be inconsistent with the *Q* matrix of the DINA model that generates data, resulting in the biased estimates and poor fitting. However, when the data are generated from MMS-DINA model, the DINA model is the worst fitting model. The worst fitting result is attributed to the relatively simple model structure, which leads to the phenomenon of under-fitting. Finally, we draw a valuable conclusion that no matter which models (DINA and MS-DINA) generate the data, the MMS-DINA is better fitting model than other not true models. However, in the real data analysis, the DINA model is preferred for this data set because its relatively simple formulation do not lead to worse fit compared with the MS-DINA model and MMS-DINA model.

Classification methods based on CDMs play an important role in cognitive diagnosis, because it is desired in some educational settings to classify examinees as masters or non-masters of multiple discrete latent attributes. In simulation study, as an illustration, we consider the MMS-DINA model is used in the situation that 500 examinees answer 20 items, indicating that it classifies few examinees correctly on all *K* skills but classifies high ability examinees almost or exactly correctly with few severe misclassification.

Because there are a large number of parameters in MMS-DINA model, we can only rely on MCMC algorithm to estimate the parameters. However, the computational burden of the MCMC algorithm becomes intensive especially when a large number of examinees or the items is considered, or a large number of the MCMC sample size is used. Therefore, it is desirable to develop a standing-alone R package associated with C++ or Fortran software for more extensive large-scale assessment program. In addition, the convergence of Bayesian algorithm need to be further investigated in the next studies. Firstly, for the PSRF value, we use a relatively relaxed 1.2 as a cutoff for determining the convergence of Bayesian estimation based on the previous literature (Brooks and Gelman, 1998; Fagua et al., 2019). In fact, we cannot decide whether 1.2 as a cutoff is really sufficient to determine the convergence. Educational psychologists have to be more careful when choosing 1.2 as a cutoff. This is because the effective sample size (ESS) can be only small, which can result in the summary statistics for the chain that provide only poor approximations of the Bayesian estimates. More specifically, the mean of the chain might not be very close to the expected value of the posterior distribution from the perspective of Bayesian point estimation. Therefore, in more substantive applications of the model, a more conservative PSRF cutoff (e.g., PSRF <1.05) should ideally be used (Gelman et al., 2014; Vehtari et al., 2019; Zitzmann and Hecht, 2019). However, if we use a more conservative criterion for the PSRF, it is unknown how long it will take to achieve a PSRF of 1.05, and it will be a great challenge for our MMS-DINA model due to the large number of unknown parameters to be estimated. In order to achieve a cutoff of 1.05 for PSRF, we need to run a longer Markov chains to achieve the required number of ESS for convergence, but this process is very time-consuming and requires a large amount of computer memory. These require us to do a lot of simulation studies in later stages to give the definite results. Secondly, we also need to further investigate whether the obtained standard errors are accurate by using the coverage rate. However, these studies are beyond the purpose of this study to analyze the different solution strategies of the examinees by constructing a MMS-DINA model.

There are several avenues for further research on multiple-strategy models. In this paper, we focus on the comparison of multiple-strategy models under the most commonly used DINA model framework, and explore the cognitive process of solving items using different strategies among examinees, without focusing on the comparison of other multiple strategy cognitive diagnostic models, such as MS high-order DINA model, or some saturated type MS CDMs which are MS generalized DINA models, or MS loglinear cognitive model and so on. As Li et al. (2016) point out, it needs to be further explored to find the most appropriate model to fit data among the numerous cognitive diagnosis models. Therefore, in the later research, we will focus on the comparison of different MS CDMs to find out the advantages, disadvantages, and application scope of each model. In addition, the different classification methods may be helpful in both item selection and final examinee classification (Xu et al., 2003; Cheng, 2009). Also, note that a strategy is merely defined by the set of attributes required by a particular approach to solving a problem. One can imagine that a strategy might instead be determined by a set of attributes as well as a procedure and sequence for using them. So depending on how the attributes are defined, this will not always be the case, and one may consider different methods of using the same attributes. In addition, in this study, we only analyze two strategies. When the number of strategies increase, the performance of our MMS-DINA model needs to be further investigated. For example, we need to investigate that whether the identification conditions are satisfied as the number of strategies increases, as well as whether the parameter estimates are recovered well. In addition, the computational efficiency may be reduced due to the large number of parameters with the increased strategies.

## Data Availability Statement

Publicly available datasets were analyzed in this study. This data can be found at: https://cran.r-project.org/web/packages/CDM/index.html.

## Author Contributions

JL and ZZ completed the writing of the article. JZ, JL, and JY provided key technical support. JZ and JL provided revisions. SS provided original thoughts. All authors contributed to the article and approved the submitted version.

## Conflict of Interest

The authors declare that the research was conducted in the absence of any commercial or financial relationships that could be construed as a potential conflict of interest.

## References

[B1] Brooks S. Gelman A. (1998). General methods for monitoring convergence of iterative simulations. J. Comput. Graph. Stat. 7, 434–455. 10.1080/10618600.1998.10474787

[B2] Casella G. George E. I. (1992). Explaining the Gibbs sampler. Am. Stat. 46, 167–174. 10.1080/00031305.1992.10475878

[B3] Chen M.-H. Shao Q.-M. Ibrahim J. G. (2000). Monte Carlo Methods in Bayesian Computation. New York, NY: Springer. 10.1007/978-1-4612-1276-8

[B4] Chen Y. Liu J. Xu G. Ying Z. (2015). Statistical analysis of Q-matrix based diagnostic classification models. J. Am. Stat. Assoc. 110, 850–866. 10.1080/01621459.2014.934827 26294801 PMC4539161

[B5] Cheng Y. (2009). When cognitive diagnosis meets computerized adaptive testing: CD-CAT. Psychometrika 74, 619–632. 10.1007/s11336-009-9123-2

[B6] Chib S. Greenberg E. (1995). Understanding the Metropolis-Hastings algorithm. Am. Stat. 49, 327–335. 10.2307/2684568

[B7] de la Torre J. (2008). An empirically-based method of Q-matrix validation for the DINA model: Development and applications. J. Educ. Measur. 45, 343–362. 10.1111/j.1745-3984.2008.00069.x

[B8] de la Torre J. (2009). DINA model and parameter estimation: a didactic. J. Educ. Behav. Stat. 34, 115–130. 10.3102/1076998607309474

[B9] de la Torre J. (2011). The generalized DINA model framework. Psychometrika 76, 179–199. 10.1007/s11336-011-9207-7

[B10] de la Torre J. Douglas J. (2004). Higher-order latent trait modelsss for cognitive diagnosis. Psychometrika 69, 333–353. 10.1007/BF02295640

[B11] de la Torre J. Douglas J. (2008). Model evaluation and multiple strategies in cognitive diagnosis: an analysis of fraction subtraction data. Psychometrika 73, 595–624. 10.1007/s11336-008-9063-2

[B12] DiBello L. Roussos L. Stout W. (2007). 31A Review of cognitively diagnostic assessment and a summary of psychometric models 1,2. Handb. Stat. 26, 979–1030. 10.1016/S0169-7161(06)26031-0

[B13] Diebold J. Robert C. P. (1994). Estimation of finite mixture distribution through Bayesian sampling. J. R. Stat. Soc. B 56, 163–175. 10.1111/j.2517-6161.1994.tb01985.x

[B14] Doignon J. P. Falmagne J. C. (1999). Knowledge Spaces. New York, NY: Springer. 10.1007/978-3-642-58625-5

[B15] Fagua J. C. Baggio J. A. Ramsey R. D. (2019). Drivers of forest cover changes in the Chocó-Darien Global Ecoregion of South America. Ecosphere 10:e02648. 10.1002/ecs2.2648 PMC6358088 30707720

[B16] Fuson K. C. Smith S. Lo Cicero A. (1997). Supporting Latino first graders' ten-structured thinking in urban classrooms. J. Res. Math. Educ. 28, 738–760. 10.2307/749640

[B17] Geisser S. Eddy W. F. (1979). A predictive approach to model selection. J. Am. Stat. Assoc. 74, 153–160. 10.2307/2286745

[B18] Gelfand A. E. Dey D. K. Chang H. (1992). Model determination using predictive distributions with implementation via sampling-based methods (with Discussion), in Bayesian Statistics 4, eds Bernado J. M. Berger J. O. Dawid A. P. Smith A. F. M. (Oxford: Oxford University Press), p. 147–167.

[B19] Gelman A. Carlin J. B. Stern H. S. Dunson D. B. Vehtari A. Rubin D. B. (2014). Bayesian Data Analysis, 3rd Edn. Boca Raton, FL: CRC Press. 10.1201/b16018

[B20] Gelman A. Rubin D. B. (1992). Inference from iterative simulation using multiple sequences. Stat. Sci. 7, 457–472. 10.2307/2246093

[B21] Geman S. Geman D. (1984). Stochastic relaxation, Gibbs distributions and the Bayesian restoration of images. IEEE Trans. Pattern Anal. Mach. Intell. 6, 721–741. 10.1109/TPAMI.1984.4767596 22499653

[B22] Gilks W. R. (1996). Full conditional distributions, in Markov Chain Monte Carlo in Practice, eds Gilks W. R. Richardson S. Spiegelhalter D. J. (Washington, DC: Chapman and Hall), 75–88. 10.1007/978-1-4899-4485-6_5

[B23] Gorin J. S. (2007). Test cnstruction and diagnostic testing, in Cognitive Diagnostic Assessment for Education: Theory and Applications, eds Leighton J. P. Gierl M. J. (New York, NY: Cambridge University Press), 173–201. 10.1017/CBO9780511611186.007

[B24] Haberman S. J. von Davier M. (2007). Some notes on models for cognitively based skill diagnosis. Handb. Stat. 26, 1031–1038. 10.1016/S0169-7161(06)26040-1

[B25] Haertel E. H. (1989). Using restricted latent class models to map the skill structure of achievement items. J. Educ. Meas. 26:21. 10.1111/j.1745-3984.1989.tb00336.x

[B26] Henson R. A. Templin J. L. Willse J. T. (2009). Defining a family of cognitive diagnosis models using log-linear models with latent variables. Psychometrika 74, 191–210. 10.1007/s11336-008-9089-5

[B27] Hung L.-F. Wang W.-C. (2012). The generalized multilevel facets model for longitudinal data. J. Educ. Behav. Stat. 37, 231–255. 10.3102/1076998611402503

[B28] Huo Y. de la Torre J. (2014). An EM algorithm for the multiple-strategy DINA model. Appl. Psychol. Meas. 38, 464–485. 10.1177/0146621614533986

[B29] Ibrahim J. G. Chen M.-H. Sinha D. (2001). Bayesian Survival Analysis. New York, NY: Springer. 10.1002/0470011815.b2a11006

[B30] Junker B. W. Sijtsma K. (2001). Cognitive assessment models with few assumptions, and connections with nonparametric item response theory. Appl. Psychol. Meas. 25, 258–272. 10.1177/01466210122032064

[B31] Lenk P. J. DeSarbo W. S. (2000). Bayesian inference for finite mixtures of generalized linear models with random effects. Psychometrika 65, 93–119. 10.1007/BF02294188

[B32] Li H. Hunter C. V. Lei P. W. (2016). The selection of cognitive diagnostic models for a reading test. Lang. Test. 33, 391–409. 10.1177/0265532215590848

[B33] Patz R. J. Junker B. W. (1999a). A straightforward approach to Markov chain Monte Carlo methods for item response theory. J. Educ. Behav. Stat. 24, 146–178. 10.3102/10769986024002146

[B34] Patz R. J. Junker B. W. (1999b). Applications and extensions of MCMC in IRT: multiple item types, missing data, and rated responses. J. Educ. Behav. Stat. 24, 342–366. 10.3102/10769986024004342

[B35] Plummer M. Best N. Cowles K. Vines K. (2006). CODA: convergence diagnosis and output analysis of MCMC. R News 6, 7–11. 10.1159/000323281

[B36] Robert C. P. (1996). Mixtures of distributions: inference and estimation, in Markov Chain Monte Carlo in Practice, eds Gilks W. R. Richardson S. Spiegelhalter D. J. (Washington, DC: Chapman & Hall), 75–88. 10.1007/978-1-4899-4485-6_24

[B37] Spiegelhalter D. J. Best N. G. Carlin B. P. Van der Linde A. (2002). Bayesian measures of model complexity and fit. J. R. Stat. Soc. Ser. B Stat Methodol. 64, 583–639. 10.1111/1467-9868.00353

[B38] Tatsuoka C. (2002). Data-analytic methods for latent partially ordered classification models. J. R. Stat. Soc. Ser. C Appl. Stat. 51, 337–350. 10.1111/1467-9876.00272

[B39] Templin J. L. Henson R. A. (2006). Measurement of psychological disorders using cognitive diagnosis models. Psychol. Methods 11, 287–305. 10.1037/1082-989x.11.3.287 16953706

[B40] Titterington D. M. Smith A. F. M. Makov U. E. (1985). Statistical Analysis of Finite Mixture Distributions. New York, NY: Wiley. 10.2307/2531224

[B41] Vehtari A. Gelman A. Simpson D. Carpenter B. Bürkner P. C. (2019). Rank-normalization, folding, and localization: an improved $\hat{R}$ for assessing convergence of MCMC. ArXiv[Preprint] 1–27. 10.1214/20-ba1221

[B42] von Davier M. (2014). The DINA model as a constrained general diagnostic model: two variants of a model equivalency. Br. J. Math. Stat. Psychol. 67, 49–71. 10.1111/bmsp.12003 23297749

[B43] Xu X. Chang H. Douglas J. (2003). A simulation study to compare CAT strategies for cognitive diagnosis. Paper Presented at the Annual Meeting of the American Educational Research Association (Chicago, IL).

[B44] Zitzmann S. Hecht M. (2019). Going beyond convergence in Bayesian estimation: why precision matters too and how to assess it. Struct. Equat. Model. 26, 646–661. 10.1080/10705511.2018.1545232

