# Supplementary material for: Exploring Multiple Strategic Problem Solving Behaviors in Educational Psychology Research by Using Mixture Cognitive Diagnosis Model
Source: Front Psychol. 2021 Jun 3;12:568348. doi: 10.3389/fpsyg.2021.568348 (PMC8211444; doi:10.3389/fpsyg.2021.568348)
Supplement: Supplementary file 1 [file Data_Sheet_1.PDF]

# Supplement B: MATLAB Codes for MCMC sampling and model assessment

## Supplement B.1: MATLAB Codes for MCMC sampling

```
for WWW=1:EM
        %draw c
         for i=1:examn
             for w=1:M
                 LL(w)=1;
                 for j=1:itemn
                     yita(w)=1;
                     for k=1:K
                         yit=alpha(i,k).^q(j,k,w);
                         yita(w)=yita(w).*yit;
                     end
                     tem=(s_c(j,w).^yita(w)).*(g(j,w).^(1-yita(w)));
                     if score(i,j)==1
                         p(w)=tem;
                     else
                         p(w)=1-tem;
                     end
                     LL(w)=LL(w).*p(w);  %% likelihood
                 end
                 L(w)=binopdf(sum(alpha(i,:),2),k_total,mu(w));
                 Lc(w)=L(w).*LL(w).*pai(w);
             end
             c_hat(i,:) = 10^5*Lc;
             pp=Lc(2)./(Lc(1)+Lc(2));
             c(i)=binornd(1,pp)+1;
             %pause
         end
         %c_hat(:,WWW,rep) = c;
         %draw alpha
            for i=1:examn
                for k=1:k_total
                    alpha_new(i,k)=binornd(1,0.5,1,1);
                end
                alpha_new;
                alpha;
                ww=c(i);
                LLa=binopdf(sum(alpha_new(i,:),2),k_total,mu(ww))
                ./binopdf(sum(alpha(i,:),2),k_total,mu(ww));

                LLLa=1;
                for j=1:itemn
                    yitt=1;
                    yita_new=1;
```

```
            for k=1:K
                yit=alpha(i,k).^q(j,k,ww);
                yitt=yitt.*yit;
                yit_new=alpha_new(i,k).^q(j,k,ww);
                yita_new=yita_new.*yit_new;
            end
             temm=(s_c(j,ww).^yitt).*(g(j,ww).^(1-yitt));
             tem_new=(s_c(j,ww).^yita_new).*(g(j,ww).^(1-yita_new));
             if score(i,j)==1
                 p=temm;
                 p_new=tem_new;
             else
                 p=1-temm;
                 p_new=1-tem_new;
             end
            temp=(p_new./p);
            LLLa=LLLa.*temp;
        end
        %pause
        p3=LLa.*LLLa;
        t=rand(1,1);
        if p3>=t
            alpha(i,:)=alpha_new(i,:);
        end
        %pause
    end
% draw s,g
    for j=1:itemn
        for w=1:M
            temp1_g=0.2;
            temp2_g=0.0;
            temp1_s_c=0.8;
            temp2_s_c=0.6;
            g_new(j,w) = randi([fix(temp2_g*1000),fix(temp1_g*1000)],1,1)/1000;
            s_c_new(j,w) = randi([fix(temp2_s_c*1000),fix(temp1_s_c*1000)],1,1)/1000;
        end
        LLb1=1;
        LLb2=1;

        alpha;
        g; s_c;
        g_new; s_c_new;

        for i=1:examn
            ww=c(i);
            if ww==1
                 yitat=1;
                 for k=1:K
                     yit=alpha(i,k).^q(j,k,ww);
```

```
                yitat=yitat.*yit;
            end
            tem=(s_c(j,ww).^yitat).*(g(j,ww).^(1-yitat));
            tem_new=(s_c_new(j,ww).^yitat).*(g_new(j,ww).^(1-yitat));
            if score(i,j)==1
                p=tem;
                p_new=tem_new;
            else
                p=1-tem;
                p_new=1-tem_new;
            end
            temp1=(p_new./p);
            LLb1=LLb1.*temp1;
        else
            yitw=1;
            for k=1:K
                yiw=alpha(i,k).^q(j,k,ww);
                yitw=yitw.*yiw;
            end
            tem=(s_c(j,ww).^yitw).*(g(j,ww).^(1-yitw));
            tem_new=(s_c_new(j,ww).^yitw).*(g_new(j,ww).^(1-yitw));
            if score(i,j)==1
                p=tem;
                p_new=tem_new;
            else
                p=1-tem;
                p_new=1-tem_new;
            end
            temp2=(p_new./p);
            LLb2=LLb2.*temp2;
        end
    end
    %pause
    t=rand(1,1);
    if LLb1>=t
        g(j,1)=g_new(j,1);
        s_c(j,1)=s_c_new(j,1);
    end
    t=rand(1,1);
    if LLb2>=t
        g(j,2)=g_new(j,2);
        s_c(j,2)=s_c_new(j,2);
    end

  end
  slipping(:,WWW,rep) = s_c(:);
  guessing(:,WWW,rep) = g(:);

%draw pai ~
```

```
            ss=sum(c)-examn;
            rr1=ss+0.01;
            rr2=examn-ss+0.01;
            dd1=gamrnd(rr1,1,1,1);
            dd2=gamrnd(rr2,1,1,1);
            pai1=dd1./(dd1+dd2);
            pai2=dd2./(dd1+dd2);
            pai=[pai1 pai2];
            pai_hat(:,WWW,rep) = pai';
            %pause
        %draw mu
            aa=sum(alpha,2);
            rrt1=0;
            rrt2=0;
            for i=1:examn
                wt=c(i);
                if wt==1
                    rrt1=rrt1+aa(i);
                else
                    rrt2=rrt2+aa(i);
                end
            end
            ddt1=rrt1+aw;
            ddt2=N*K+bw-rrt1;
            dd3=rrt2+aw;
            dd4=N*K+bw-rrt2;
            mu1=betarnd(ddt1,ddt2);
            mu2=betarnd(dd3,dd4);
            mu=[mu1 mu2];
           %pause
            if WWW>=EM-BI+1
                 vvv=vvv+1;
                 for i=1:examn
                     for k=1:k_total
                         alpha_alpha(i,k)=alpha_alpha(i,k)+alpha(i,k);
                     end
                 end
                 for j=1:itemn
                     for w=1:M
                         s_c_s_c(j,w)=s_c_s_c(j,w)+s_c(j,w);
                         g_g(j,w)=g_g(j,w)+g(j,w);
                     end
                 end
            end
End
```

## Supplement B.2: Matlab Codes for Computing DIC and LPML

```
for rep = 1:repetition
    for WWW=1:EM
        %draw c
         for i=1:examn
             for w=1:M
                 LL(w)=1;
                 for j=1:itemn
                     yita(w)=1;
                     for k=1:K
                         yit=alpha(i,k).^q(j,k,w);
                         yita(w)=yita(w).*yit;
                     end
                     tem=(s_c(j,w).^yita(w)).*(g(j,w).^(1-yita(w)));
                     if score(i,j)==1
                         p(w)=tem;
                     else
                         p(w)=1-tem;
                     end
                     LL(w)=LL(w).*p(w);  %% likelihood
                 end
                 L(w)=binopdf(sum(alpha(i,:),2),k_total,mu(w));
                 Lc(w)=L(w).*LL(w).*pai(w);
             end
             pp=Lc(2)./(Lc(1)+Lc(2));
             c(i)=binornd(1,pp)+1;
             %pause
         end
         %draw alpha
            for i=1:examn
                for k=1:k_total
                    alpha_new(i,k)=binornd(1,0.5,1,1);
                end
                alpha_new;
                alpha;
                ww=c(i);
               LLa=binopdf(sum(alpha_new(i,:),2),k_total,mu(ww))
                 ./binopdf(sum(alpha(i,:),2),k_total,mu(ww));
                LLLa=1;
                for j=1:itemn
                    yitt=1;
                    yita_new=1;
                    for k=1:K
                        yit=alpha(i,k).^q(j,k,ww);
                        yitt=yitt.*yit;
                        yit_new=alpha_new(i,k).^q(j,k,ww);
                        yita_new=yita_new.*yit_new;
                    end
```

```
          temm=(s_c(j,ww).^yitt).*(g(j,ww).^(1-yitt));
       tem_new=(s_c(j,ww).^yita_new).*(g(j,ww).^(1-yita_new));
          if score(i,j)==1
              p=temm;
              p_new=tem_new;
          else
              p=1-temm;
              p_new=1-tem_new;
          end
         temp=(p_new./p);
         LLLa=LLLa.*temp;
     end
     %pause
     p3=LLa.*LLLa;
     t=rand(1,1);
     if p3>=t
         alpha(i,:)=alpha_new(i,:);
     end
     %pause
 end
 % draw s,g
 for j=1:itemn
     for w=1:M
     temp1_g=0.3;
     temp2_g=0.1;
     temp1_s_c=0.9;
     temp2_s_c=0.7;
g_new(j,w)=randi([fix(temp2_g*1000),fix(temp1_g*1000)],1,1)/1000;
s_c_new(j,w)=randi([fix(temp2_s_c*1000),fix(temp1_s_c*1000)],1,1)/1000;
     end
     LLb1=1;
     LLb2=1;

     alpha;
     g; s_c;
     g_new; s_c_new;

     for i=1:examn
         ww=c(i);
         if ww==1
              yitat=1;
              for k=1:K
                  yit=alpha(i,k).^q(j,k,ww);
                  yitat=yitat.*yit;
              end
              tem=(s_c(j,ww).^yitat).*(g(j,ww).^(1-yitat));
    tem_new=(s_c_new(j,ww).^yitat).*(g_new(j,ww).^(1-yitat));
              if score(i,j)==1
                  p=tem;
```

```
                p_new=tem_new;
            else
                p=1-tem;
                p_new=1-tem_new;
            end
            temp1=(p_new./p);
            LLb1=LLb1.*temp1;
        else
            yitw=1;
            for k=1:K
                yiw=alpha(i,k).^q(j,k,ww);
                yitw=yitw.*yiw;
            end
            tem=(s_c(j,ww).^yitw).*(g(j,ww).^(1-yitw));
     tem_new=(s_c_new(j,ww).^yitw).*(g_new(j,ww).^(1-yitw));
            if score(i,j)==1
                p=tem;
                p_new=tem_new;
            else
                p=1-tem;
                p_new=1-tem_new;
            end
            temp2=(p_new./p);
            LLb2=LLb2.*temp2;
        end
    end
    %pause
    t=rand(1,1);
    if LLb1>=t
        g(j,1)=g_new(j,1);
        s_c(j,1)=s_c_new(j,1);
    end
    t=rand(1,1);
    if LLb2>=t
        g(j,2)=g_new(j,2);
        s_c(j,2)=s_c_new(j,2);
    end

  end
  slipping(:,WWW,rep) = s_c(:);
  guessing(:,WWW,rep) = g(:);

%draw pai
  ss=sum(c)-examn;
  rr1=ss+0.01;
  rr2=examn-ss+0.01;
  dd1=gamrnd(rr1,1,1,1);
  dd2=gamrnd(rr2,1,1,1);
  pai1=dd1./(dd1+dd2);
```

```
        pai2=dd2./(dd1+dd2);
        pai=[pai1 pai2];
        %pause
    %draw mu
        aa=sum(alpha,2);
        rrt1=0;
        rrt2=0;
        for i=1:examn
            wt=c(i);
            if wt==1
                rrt1=rrt1+aa(i);
            else
                rrt2=rrt2+aa(i);
            end
        end
        ddt1=rrt1+aw;
        ddt2=N*K+bw-rrt1;
        dd3=rrt2+aw;
        dd4=N*K+bw-rrt2;
        mu1=betarnd(ddt1,ddt2);
        mu2=betarnd(dd3,dd4);
        mu=[mu1 mu2];
       %pause
        if WWW>=EM-BI+1
             vvv=vvv+1;
             for i=1:examn
                 for k=1:k_total
                     alpha_alpha(i,k)=alpha_alpha(i,k)+alpha(i,k);
                 end
             end
             for j=1:itemn
                 for w=1:M
                     s_c_s_c(j,w)=s_c_s_c(j,w)+s_c(j,w);
                     g_g(j,w)=g_g(j,w)+g(j,w);
                 end
             end
        end
end

Alpha=(alpha_alpha)./BI;
S_C=(s_c_s_c)./BI;
S=1-S_C;
G=(g_g)./BI;
for i=1:examn
    for k=1:k_total
        if Alpha(i,k)>=0.5
            Alpha2(i,k)=1;
        else
            Alpha2(i,k)=0;
```

```
            end
        end
    end
%% s g
    guessing_hat = mean(guessing(:,(BI+1):end,rep),2);
    slipping_hat = mean(slipping(:,(BI+1):end,rep),2);
    eta = zeros(person,item,strategy); eta_temp = zeros(person,item);
    p_MMS = zeros(person,item);
    p_MMS_temp = zeros(person,item,strategy);
    % mixture multiple strategy (MMS) model
    for m = 1:strategy
        eta(:,:,m) = eta_(alpha,q(:,:,m));
    p_MMS_temp(:,:,m) = ((ones(person,1) * slipping_hat(item*(m-1)+1:item*m)') .^ eta(:,:,m))
    .* ((ones(person,1) * guessing_hat(item*(m-1)+1:item*m)') .^ (1 - eta(:,:,m)));
    p_MMS = p_MMS + pai(m) * (p_MMS_temp(:,:,m).^score.*(1-p_MMS_temp(:,:,m)).^(1-score));
    end
    p_MMS_log(:,:,rep) = log(p_MMS);  logLik_MMS(rep) = sum(sum(log(p_MMS)));
    % DINA model
    p_DINA_1 = p_MMS_temp(:,:,1);  p_DINA_2 = p_MMS_temp(:,:,2);
    p_DINA_1_log(:,:,rep) = score.*log(p_DINA_1) + (1-score).*log(1-p_DINA_1);
    logLik_DINA_1(rep) = sum(sum(p_DINA_1_log(:,:,rep)));
    p_DINA_2_log(:,:,rep) = score.*log(p_DINA_2) + (1-score).*log(1-p_DINA_2);
    logLik_DINA_2(rep) = sum(sum(p_DINA_2_log(:,:,rep)));
    % multiple strategy (MS) model
    for i = 1:person
        for j = 1:item
            if (eta(i,j,1)-eta(i,j,2) >= 0)
                eta_temp(i,j) = eta(i,j,1);
            else
                eta_temp(i,j) = eta(i,j,2);
            end
        end
    end
    p_MS_1 = ((ones(person,1) * slipping_hat(1:item)') .^ eta_temp)
    .* ((ones(person,1) * guessing_hat(1:item)') .^ (1 - eta_temp));          %% strategy 1
    p_MS_2 = ((ones(person,1) * slipping_hat(item+1:2*item)') .^ eta_temp)
     .* ((ones(person,1) * guessing_hat(item+1:2*item)') .^ (1 - eta_temp));  %% strategy 2
    p_MS_1_log(:,:,rep) = score.*log(p_MS_1) + (1-score).*log(1-p_MS_1);
    logLik_MS_1(rep) = sum(sum(p_MS_1_log(:,:,rep)));
    p_MS_2_log(:,:,rep) = score.*log(p_MS_2) + (1-score).*log(1-p_MS_2);
    logLik_MS_2(rep) = sum(sum(p_MS_2_log(:,:,rep)));
end
%% DIC
    DIC_DINA_1 = 2 * (-2*mean(logLik_DINA_1)) - (-2*max(logLik_DINA_1));
    DIC_DINA_2 = 2 * (-2*mean(logLik_DINA_2)) - (-2*max(logLik_DINA_2));
    DIC_MS_1 = 2 * (-2*mean(logLik_MS_1)) - (-2*max(logLik_MS_1));
    DIC_MS_2 = 2 * (-2*mean(logLik_MS_2)) - (-2*max(logLik_MS_2));
    DIC_MMS = 2 * (-2*mean(logLik_MMS)) - (-2*max(logLik_MMS));
%%  CPO(LPML) conditional predictive ordinate
```

```
logCPO_DINA_1 = - log(sum(exp(-p_DINA_1_log),3)/repetition);
logCPO_DINA_2 = - log(sum(exp(-p_DINA_2_log),3)/repetition);
logCPO_MS_1 = - log(sum(exp(-p_MS_1_log),3)/repetition);
logCPO_MS_2 = - log(sum(exp(-p_MS_2_log),3)/repetition);
logCPO_MMS = - log(sum(exp(-p_MMS_log),3)/repetition);
LPML_DINA_1 = sum(sum(logCPO_DINA_1));
LPML_DINA_2 = sum(sum(logCPO_DINA_2));
LPML_MS_1 = sum(sum(logCPO_MS_1));
LPML_MS_2 = sum(sum(logCPO_MS_2));
LPML_MMS = sum(sum(logCPO_MMS));
```
